# Self‐Determination in Action: A Scoping Review on Oral Health Training for Indigenous Health Workers Globally

**DOI:** 10.1111/cdoe.70009

**Published:** 2025-08-10

**Authors:** Madison Cachagee, Brianna Poirier, Fernanda Doak, Sneha Sethi, Joanne Hedges, Michael Larkin, Lisa Jamieson

**Affiliations:** ^1^ Indigenous Oral Health Unit, Australian Research Centre for Population Oral Health, Adelaide Dental School, the University of Adelaide Adelaide South Australia Australia; ^2^ Adelaide Medical School, the University of Adelaide Adelaide South Australia Australia

**Keywords:** community health workers, health inequities, health promotion, indigenous peoples, oral health

## Abstract

Globally, the systemic marginalisation of Indigenous Peoples has led to significant health disparities rooted in the legacy of colonisation and ongoing settler colonialism.

**Objective:**

This scoping review aimed to collate oral health promotion training programmes tailored for Indigenous Health Workers (IHW), who play a pivotal role in improving health outcomes by bridging mainstream healthcare with Indigenous Communities, globally.

**Methods:**

A systematic, two‐step search was conducted across five databases—PubMed, Scopus, Web of Science, EMBASE and ProQuest Central—without geographic restrictions. Two independent reviewers screened studies, and additional sources were identified from reference lists. A supplementary search of grey literature was performed in Google Scholar and relevant websites.

**Results:**

Of the 374 eligible articles, 11 programmes fulfilled the inclusion criteria. These programmes covered 10 topic areas, including: oral anatomy, early childhood oral health, and the influence of diet and chronic disease on oral health. Effective programmes were collaboratively developed with Indigenous Communities, aligning closely with Community needs and promoting self‐determination. The findings emphasise the importance of involving IHW in oral health initiatives to tackle ongoing oral disease disparities and advance oral health equity for Indigenous populations.

**Conclusion:**

By prioritising Indigenous leadership and cultural knowledge, these programs exemplify avenues for strengthening equitable, culturally centred healthcare for Indigenous Communities globally. There remains a critical need for sustainable investment in IHW oral health training, enabling Indigenous‐led initiatives to meaningfully address oral health disparities.

## 1 Introduction

Indigenous (throughout this paper, Indigenous is used as a global, collective term to refer to the First Peoples of various lands, unless otherwise specified. Where appropriate, more specific nation, group or community names are used to respect local contexts and identities) Peoples face a disproportionate burden of early death and severe, chronic illness compared to their settler counterparts—an enduring and pervasive reality across settler colonial nations. This stark health inequity persists despite centuries of resistance against colonisation's efforts to erase Indigenous existence and identity [1, 2, 3]. While colonisation initially focused on resource and labour exploitation, the primary goal of settler colonialism is to maintain control over Indigenous lands to permanently occupy them. In doing so, settlers aim to eliminate Indigenous Communities—not just as individuals, but also as distinct groups with sovereign rights and claims to their land. The ultimate objective is to erase the presence and authority of Indigenous Peoples in order to assert full control over the land [4]. Colonial societies are inherently structured to prioritise the needs of settlers, systematically oppressing colonised peoples in the process. This belief in the superiority of (White) settlers provided the foundation of colonial health systems, which were established on the premise that colonised peoples are inferior. As a result, these health systems and their services, which are often imposed on Indigenous Communities, are understaffed, underfunded, and do not support comprehensive health and wellbeing for Indigenous Peoples. The resulting and persisting health inequities experienced by Indigenous Peoples, due to the inadequacies of health systems, serve as justification for further colonial control, reinforcing existing social and power hierarchies. Ultimately, rather than addressing underlying inequities created by colonisation, health systems perpetuate and sustain a discourse of necessary colonial dominance [4].

Driven by the critical need for self‐determination and the persistent failures of mainstream health systems, Indigenous Communities worldwide have continually taken matters into their own hands, a resistance that has been in motion since the earliest days of colonisation. These efforts are premised on the fundamental pillars of Indigenous wellbeing (connections, relationships and family) that are vital components shaping physical, emotional and spiritual health, with land playing a central role in overall wellbeing [5, 6]. This inherently holistic perspective encompasses mental, physical, cultural, and spiritual dimensions and extends beyond the notion of the ‘whole body’ to embrace the interconnected relationships that underpin cultural wellbeing. These include spiritual, environmental, ideological, political, social, economic, mental, and physical factors all working together in harmony, rather than being influenced by colonial dominance [6]. In so‐called Australia (the use of the term ‘so‐called Australia’ acknowledges the colonial imposition on Indigenous lands and the fact that sovereignty was never ceded. It reflects critical scholarship that challenges the legitimacy of the colonial state and recognises the enduring sovereignty and presence of Aboriginal and Torres Strait Islander Peoples), this has led to the establishment of Aboriginal Community Controlled Health Services (ACCHS), which effectively bridge the gaps left by colonial health systems and are accountable to the Communities they serve [7]. These services are locally governed and operated by Aboriginal Communities, delivering holistic, comprehensive, and culturally appropriate care that supports the social, emotional, physical, and cultural wellbeing of their people [8]. Similarly, on Turtle Island, the Six Nations of the Grand River in Ontario, Canada have established a groundbreaking palliative care programme specifically designed to reflect their Community, culture, and regional context on their lands [9].

Indigenous health workers (IHW) play a crucial role in improving health outcomes within these settings for Indigenous populations worldwide [7, 10, 11, 12, 13, 14]. The impact of IHW spans a broad range of efforts, from health promotion initiatives to disease management [7]. These workers, who are members of their own communities, serve as essential bridges between mainstream health systems and Indigenous cultures [10, 15]. The trust IHW build with patients and the strong relationships they uphold within their cultural contexts are invaluable resources [11]. In Australia, IHW are supported through nationally accredited training programmes, such as the Aboriginal and Torres Strait Islander Primary Health Care Certificates and Diploma. A similar initiative exists within the United States through the Community Health Representative Diploma, offered by Indian Health Services [16]. Both programmes equip health workers with the skills to provide holistic, culturally relevant care across a range of health domains.

However, the Australian context is unique in that Aboriginal and Torres Strait Islander Health Practitioners are the only nationally registered, culturally based health profession, governed by the National Association of Aboriginal and Torres Strait Islander Health Workers and Practitioners (NAATSIHWP) [17]. While IHW programmes are present in both Australia and the United States, the Aboriginal and Torres Strait Islander Primary Health Care Certificates and Diploma is distinct in that oral health is offered as a non‐mandatory elective within the programme. This highlights how the involvement of IHW in oral health promotion and care delivery remains inconsistent and poorly defined [13, 14]. This underutilisation is driven by several factors, including limited access to oral health training, the perception of oral health as a specialised field, and persistent colonial and racist attitudes that undervalue IHW contributions to mainstream health services. However, integrating non‐dental practitioners, particularly IHW, into the dental workforce is of paramount importance for achieving greater equity in oral health outcomes [7].

Given the critical role of IHW in supporting Community well‐being [7, 10, 11, 12, 13, 14] and the persistent oral health disparities faced by Indigenous populations worldwide [14, 18], it is evident that these health inequities, including oral health, are not merely coincidental. Rather, they are deeply rooted in the historical and ongoing impacts of colonial health systems, which have systematically marginalised Indigenous perspectives, knowledge and practices in oral health care delivery [19]. Indigenous Communities face significant and unacceptable oral health inequities, with access to essential dental care being just one of many critical areas that are consistently neglected worldwide [18]. The provision of culturally unsafe care, constant experiences of racism, and dental anxiety rooted in historical mistreatment are just a few of the formidable barriers Indigenous Peoples face when accessing mainstream dental services [20]. Therefore, this scoping review aimed to comprehensively examine the landscape of oral health promotion training tailored for IHW on a global scale. By systematically exploring existing literature, this study has identified and analysed training programmes, initiatives, and educational resources that have been developed to equip IHW with oral health promotion skills. By focusing on the intersection of IHW and oral health promotion, this review aimed to examine and highlight an often overlooked but crucial aspect of Indigenous healthcare and pave the way for more equitable and effective oral health interventions.

## 2 Methods

This scoping review protocol has been made publicly available on Open Science Framework [21] and is in accordance with the methodological guidelines of the Joanna Briggs Institute [22]. No similar studies either registered or published were identified through a search of PubMed and PROSPERO. This review is reported in alignment with the Preferred Reporting Items for Systematic Reviews and Meta‐Analyses guidelines [23, 24] (Appendix S1).

### 2.1 Positionality Statement

In recognition of the deeply relational nature of Indigenous research, we intentionally position ourselves within this work, acknowledging our responsibilities, connections, and the relationships that shape and inform our approach [25, 26, 27]. We are guided by a proud and culturally connected Mushkegowuk woman, who leads with the strength of her identity as a daughter, sister, auntie, and researcher [28]. Our team includes both Indigenous and non‐Indigenous members from various regions: northern Turtle Island (colonially known as Canada), Australia, Brazil, India and Aotearoa/New Zealand. Our team brings together diverse perspectives to confront racial inequities, advance decolonisation, and prioritise Indigenous health. We approach this work with deep humility and gratitude, sharing the immense honour of collaborating with Indigenous Communities. Our partnerships, spanning numerous projects, are grounded in a collective dedication to supporting the self‐determination and well‐being of Indigenous Peoples. We are committed to continuous learning and growth throughout this ongoing journey.

### 2.2 Theoretical Foundations

This scoping review is firmly grounded in decolonising methodologies, critically interrogating the ongoing impacts of colonisation and the systemic exclusion of those whose identities, experiences, and values challenge colonial frameworks and dominant cultural norms [29]. Through this approach, the review actively confronts and dismantles colonial power structures, positioning Indigenous voices and knowledge systems at its core [30]. Employing Indigenous methodologies, this review not only acknowledges but centres Indigenous ways of knowing, which prioritise relationality, deep community engagement, and a holistic approach to understanding [29, 30].

A crucial component of this research was the commitment to rigorous, individual, and collective reflection among the team. This process allowed for extensive engagement with the literature, while transformative dialogues encouraged team members to examine their own positionalities and biases [31]. Furthermore, the review places strong emphasis on amplifying Indigenous voices through active and ongoing Community involvement, ensuring that the perspectives and lived experiences of Indigenous Peoples remain integral to the research process [32]. By centring these Indigenous perspectives, this scoping review aspires to generate impactful and credible findings that advance the dialogue on decolonisation in research, contributing to a future where Indigenous knowledge is recognised and valued [31, 32].

### 2.3 Identifying Articles for Inclusion

A two‐step search strategy was employed for this review. Initially, five databases (PubMed, Scopus, Web of Science, EMBASE and ProQuest Central) were searched from the inception of each database to June 7, 2024, using keywords and index terms related to ‘Indigenous’, ‘Health worker’, ‘Oral Health Promotion’ and ‘Training’ (Population). The search strategy, including all identified keywords and index terms was adapted for each included database, and the search was not limited by language, location, study design or publication date (Context) (Appendix S2). To be included in this review, articles needed to discuss oral health training programs developed for IHW (Concept). Studies were eligible if they specifically focused on oral health training for IHW, with a clear emphasis on Indigenous Community involvement in the design or implementation of the program. Excluded were studies that did not target Indigenous Communities, did not involve training, or lacked primary data. Studies were excluded if they were opinion pieces, editorials, or did not present original research. The reference lists of included sources were also screened for additional studies. Records identified during the database search were compiled into Covidence (https://www.covidence.org/, Veritas Health Innovation Ltd., Melbourne, Australia). After the removal of duplicates, two independent reviewers completed title and abstract screening against the inclusion criteria. Articles deemed relevant by either reviewer were moved on to full‐text review, where the full texts were assessed against the inclusion criteria. Any disagreements during the screening process were resolved through discussion with a third reviewer. In line with scoping review methodologies, no critical appraisal was performed, as the goal was to compile existing evidence on oral health training programs for IHW, rather than to produce critically appraised findings [22].

As a second step, a grey literature search was conducted using Google Scholar and relevant websites (Informit, Australian Indigenous Health InfoNet, Indian Health Services, First Nations Health Authority, Manatu Hauora Ministry of Health, Health New Zealand Te Whatu Ora, Te Aka Whai Ora—Maori Health Authority, Alaska Native Tribal Health Consortium, Brazilian Ministry of Health's database) and unindexed journals (International Journal of Indigenous Health, Indigenous Policy Journal, Turtle Island Journal of Indigenous Health, Journal of Indigenous Wellbeing). The search strategy included a combination of the following groups of terms: (1) ‘Indigenous health worker’ OR ‘Aboriginal health worker’ OR ‘Aboriginal health practitioner’, (2) ‘Oral Health’ and (3) ‘Training’. The search was limited to the first 100 results based on the relevance of content returned by Google's PageRank algorithm, which prioritises the most relevant results. Content beyond the first 100 results tends to become more general and less specific, making it impractical to screen exhaustively [33] (Appendix S2).

### 2.4 Data Extraction and Synthesis

Data from included studies were meticulously extracted using a piloted extraction form by two independent reviewers. This comprehensive data extraction encompassed key elements such as study aims, participant characteristics, Indigenous Community involvement, training program and workshop contexts, core competency areas, levels of Community engagement and leadership, methodologies employed, and significant findings. The contextual details of the programs and workshops, along with the identified core competency areas articulated by the authors, were synthesised to form a cohesive understanding of the range of content and delivery modalities utilised by programs [34]. Employing principles of content analysis, this process aimed to uncover patterns within the data while consciously avoiding the imposition of reviewer biases. Instead, it prioritised the authentic voices of both the authors and participants [34].

## 3 Results

### 3.1 Search Results

**FIGURE 1:**
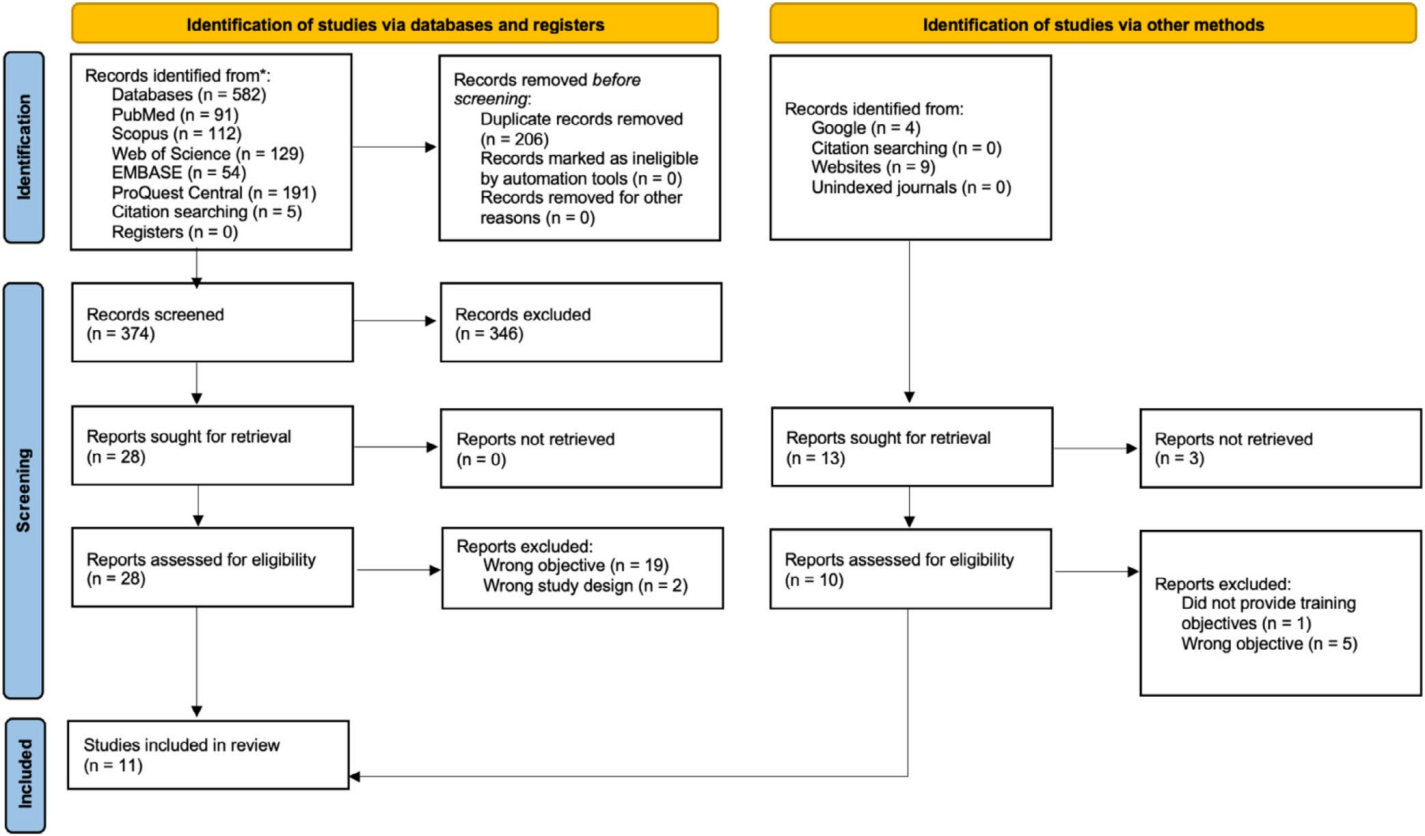
PRISMA flowchart.

The search identified 582 records; 206 records were removed due to duplication, which left 374 records for screening against inclusion criteria. Following title and abstract screening, 28 full‐text articles were retrieved and assessed. Twenty‐one articles did not fulfil inclusion criteria. Four programmes were identified through searching Google and Indigenous health services websites. Therefore, a total of 11 programmes were included in this review (Figure 1).

### 3.2 Programs' Characteristics

The programs spanned four countries, with seven from Australia [20, 35, 36, 37, 38, 39, 40], three from Turtle Island (two from United States [41, 42] and one from Canada [43]) and one from Brazil [44]. The oral health promotion training was co‐designed with various Indigenous Communities, including Aboriginal and Torres Strait Islander Peoples [20, 35, 36, 37, 38, 39, 40], Alaska Natives including Tlingit, Haida, Tsimshian [41], Navajo [42], First Nations, Inuit [43], and Metyktire (Kayapo) [44] Peoples. Seven of these programs focused on oral health promotion for the overall Indigenous Community [35, 36, 38, 40, 41, 42, 44], while two programs focused on children aged 4 years and older [37, 43], and two specifically supported pregnant mums and bubs [20, 39].

Community involvement was pivotal to the success of the reviewed oral health training programmes. Across the programmes, the nature and depth of involvement varied–ranging from consultation [35, 36, 37, 38, 42, 44] to local leadership [40, 41], governance structures [20, 39], and the inclusion of Indigenous staff in programme delivery [43]. Whether community‐driven or shaped through extensive local input, these approaches empowered community members to actively participate in decision‐making processes. This responsiveness to local needs contributed to both the cultural safety and sustainability of the programmes as evidenced by Isobelle, an AHW/P in Australia: ‘I think it's [training] really great. Honestly, the information that you've given us isn't mind blowing. So it's not like overly technical or anything. It's just like, oh damn, I didn't know that which is really good, so it's easy to understand’ [20] (Table 1).

**TABLE 1:** Characteristics of oral health promotion training programs for indigenous health workers.

Author	Title	Year	Country	Indigenous community/ies	Aim	Main outcome/results
Spetz et al.	Alaska's Primary Dental Health Aides: Adapting a Community Health Worker Program to Preventive Dental Care	2021	United States	Tlingit, Haida, Tsimshian, and other Native people within the 30 000 mile^2^ of Southeast Alaska	To address severe oral health access problems, Alaskan Native Communities and Tribal Health dentists have developed and refined an innovative workforce program over the past 15 years	Education was provided to Primary Dental Health Aides ensuring that care is delivered by individuals who share the customs and traditions of the patient population
First Nations and Inuit Health Branch, Health Canada	Generations of Smiles—COHI Aide Training Manual	2005	Canada	Inuit, First Nations	To prevent and control tooth decay in young First Nations and Inuit (FN/I) children and to set the stage for a lifetime of healthy teeth	Not applicable
Kong et al.	Aboriginal Health Workers Promoting Oral Health among Aboriginal and Torres Strait Islander Women during Pregnancy: Development and Pilot Testing of the Grinnin' Up Mums & Bubs Program	2021	Australia	Aboriginal and Torres Strait Islander	To develop an evidence‐based, culturally appropriate oral health model of care for pregnant women. To pilot test the model of care with the Aboriginal Health Workers to identify the acceptability and satisfaction of the model of care, any improvements in their oral health knowledge and confidence, and future recommendations	The training showed some improvement in oral health knowledge and confidence. The participants recommended strategies for discussing oral health with Aboriginal and Torres Strait Islander pregnant women, and changes in public health dental policy to ensure that all women would be able to access affordable dental services through the referral pathway
Meihubers	The Bila Muuji Oral Health Promotion Partnership	2013	Australia	Aboriginal and Torres Strait Islander	To identify and implement oral health promotion priorities and activities across the Bila Muuji region	These interventions continue and have become embedded in the routine activities of local ACCHS, health, and community staff
Pacza et al.	Development of oral health training for rural and remote Aboriginal health workers	2001	Australia	Aboriginal and Torres Strait Islander	To institute a culturally appropriate basic preventative oral health delivery program at a community level	The vast majority (96%) considered the material relevant to their needs. The more detailed questionnaire found that of the basic objectives set for the modules (as detailed in the learning objectives) almost all students considered they developed a good or very good understanding of these objectives
Quissell et al.	Preventing Caries in Preschoolers: Successful Initiation of an Innovative Community‐Based Clinical Trial in Navajo Nation Head Start	2014	United States	Navajo Nation	To test the effectiveness of a community‐based oral health promotion and preventive service delivery model for a reservation population	Outcome assessment includes annual dental screening and an annual caregiver survey of knowledge, attitudes and behaviours
Smith et al.	Results of a two‐year dental health education program to reduce dental caries in young Aboriginal children in New South Wales, Australia	2018	Australia	Aboriginal and Torres Strait Islander	To assess the effectiveness of a dental health education program, ‘Smiles not Tears’ in preventing Early Childhood Caries in young Aboriginal children	The value of expanding the role of AHW's in promoting oral health is that Aboriginal families have regular contact with AHW's, so dental advice can be issued and followed up regularly
Smith et al.	User assessment of an early childhood oral health education training course for Aboriginal Health Workers	2016	Australia	Aboriginal and Torres Strait Islander	To evaluate the training course in terms of its components, cultural appropriateness, course content and whether the participants felt competent to offer oral health advice.	Most AHWs thought the components of the training were very good to good including the PowerPoint presentation, graphics and materials. All thought the training and materials were culturally appropriate.
Dental Health Services Victoria	Bigger Better Smiles	2014	Australia	Aboriginal and Torres Strait Islander	To equip staff working in Aboriginal Controlled Community Health Organisations with the knowledge, skills and confidence to include oral health promotion in service provision with a focus on pregnancy and early childhood (children aged 0–3 years).	The evaluation of the pilot confirmed that the program is relevant and appropriate and showed an improvement in oral health knowledge
National Health Foundation (FUNASA), Ministry of Health (Fundacao Nacional da Saude (Funasa), Ministerio da Saude)	Professional Education Module for Indigenous Health Agents: Promoting Womens Health, Children's Health and Oral Health	2005	Brazil	Metyktire (Kayapo)	To enable Indigenous Health Agents to work in their Communities by identifying health issues in various stages of their biological cycles, developing health promotion strategies in Women, Children and Oral health, aiming at early detection of populational risk	Not specified
Kimberley Dental Team	A teaching guide for Health Care Workers	2012	Australia	Aboriginal and Torres Strait Islander	Not specified	Not applicable

### 3.3 Program Delivery

Various teaching modalities were utilised across the training programmes. Roleplay [38] activities allowed learners to practise real‐world scenarios, while 3D models provided visual aids to illustrate key concepts. Training workbooks [20, 43] and modules [36] supported individual learning. Face‐to‐face workshops facilitated interactive discussions [20, 35, 42], and PowerPoint presentations highlighted essential information [20, 38]. Group discussions further enriched the experience by encouraging collaboration and knowledge sharing among participants [38].

### 3.4 Program Content

The synthesis of program content from the 11 training programs identified ten key topic areas: oral anatomy, early childhood oral health, tooth decay, gum disease, the relationship between pregnancy and oral health, diet and sugar intake, tooth brushing techniques, dental visits, oral health assessments, and the medical conditions that influence dental treatments.

#### 3.4.1 Early Childhood Oral Health

Training programs promoted early childhood oral health through community workshops and personalised consultations with parents. One program organised hands‐on demonstrations to help parents understand tooth brushing techniques for their children. This interactive approach not only engaged caregivers but also boosted their confidence in teaching good oral hygiene practices from a young age [38]. Recognising the important role caregivers play in oral health, another program launched a caregivers' night dedicated to discussing the importance of primary teeth. During this event, participants explored strategies for preventing tooth decay, learned about the serious consequences of untreated dental issues, and understood their roles in encouraging good oral hygiene practices. By sharing knowledge across entire kinship systems, a ripple effect of positive behaviour change within families and wider communities is initiated [42].

#### 3.4.2 Oral Health During Pregnancy

Pregnant women were actively engaged through yarning sessions that highlighted the critical importance of maintaining oral health during pregnancy. These discussions were supplemented with informative take‐home resources, such as brochures and eye‐catching fridge magnets, which served as constant reminders of essential oral health practices. By making vital information easily accessible and visually prominent in participants' homes, this equips individuals to take charge of their oral health [20]. A program based in New South Wales, Australia, established a culturally safe and accessible dental service pathway specifically designed for pregnant women. By tailoring services to meet the needs of Aboriginal women, the program dismantled barriers to care and cultivated trust and community engagement [20].

#### 3.4.3 Tooth Brushing and Hygiene Practices

Addressing tooth brushing habits directly, one programme implemented supervised tooth brushing with fluoride toothpaste during school breakfast clubs and preschool activities. This hands‐on approach not only ensured that children practised effective brushing techniques but also promoted a fun, communal environment for learning about oral hygiene [35]. One programme also provided community members with toothbrushes and toothpaste [42].

#### 3.4.4 Integration of Oral Health Care

One program demonstrated innovation by integrating oral health checks and essential follow‐up dental care into an existing initiative. This integration enhanced the program's overall effectiveness and reach, ensuring that participants received comprehensive care that addressed both preventive measures and necessary treatments [35]. The amount of detail across key topics varied considerably; Table 2 provides a summary of the specific content addressed under each topic area, as reported in the included training programs.

**TABLE 2:** Description of key oral health promotion topics.

Key topic	Program content
Oral Anatomy	– Deciduous teeth and permanent teeth. – Types of teeth (incisors, canines, premolars, molars). – Tooth anatomy (crown, root). – Tooth structure (dentine, enamel, pulp). – Periodontal tissues (cemetum, periodontal ligament, alveolar bone). – Information on teeth and nerves, what causes pains, where it can radiate, how it effects you're eating ability
Early childhood oral health	– Early childhood caries. – Dummies. – Baby bottle use and tooth friendly drinks. – Tooth eruption. – Caring for children's teeth. – Thumb/finger sucking. – Ongoing health effects from dental infections in early childhood
Tooth decay	– Disease process and factors involved. – The role of fluoride and saliva. – Why decay causes toothache. – Prevention
Gum disease	– Tartar, plaque and calculus. – Disease processes and factors involved. – The role of fluoride and saliva. – Prevention
Pregnancy and oral health	– Oral health can affect baby. – Dental treatment is important and safe
Diet and sugar intake	– Sugar intake. – Fluid intake. – Types of sugar. – Hidden sugars (alcohol, coffee, energy drinks, soft drinks, tea). – Frequency of eating
Toothbrushing	– Supervised toothbrushing. – Fluoride toothpaste. – Brushing and flossing
Dental visits	– Visiting the dentist. – Fluoride application. – Dental trauma management. – Teeth extractions
Oral health checks	– ‘Lift the lip’. – Basic screening assessment
Medical conditions influencing dental treatment	– Pregnancy. – Diabetes. – Rheumatic fever and heart disease. – High blood pressure. – Renal failure. – Dialysis transplants. – Hepatitis

## 4 Discussion

This scoping review reveals a critical gap in standardised, culturally relevant oral health promotion training for IHW, drawing from 11 programs across four countries. These programs targeted oral health initiatives for Indigenous populations [35, 36, 38, 40, 41, 42, 44], with some focusing on children aged four and older [37, 43], as well as pregnant mums and bubs [20, 39]. We understand that oral health promotion training is not uniform; although we identified 11 programs, there is significant variation across different Communities and Indigenous health services globally. Despite these variations, the programs largely reflected the contexts and needs of the Communities they were implemented in, suggesting the scope of training was broadly fit for purpose. However, regional disparities in access to oral health services, resources, and funding were evident, influencing program delivery and sustainability. It is crucial that these programs are not confined solely to research settings but are actively integrated into practice. By equipping IHW with training tailored to fit with Community knowledge, values, and local health service models, they can enhance oral health outcomes and empower their Communities to thrive.

Colonial health systems have systemically marginalised Indigenous perspectives, perpetuating significant inequities in oral health [4, 45]. Indigenous Community members have indicated feeling unsafe and unwelcome in mainstream dental services, largely due to an extensive lack of cultural understanding, respect, and the lingering fear stemming from experiences of racism under colonial health system frameworks [46, 47]. This harsh reality reinforces the urgent need for culturally safe and anti‐racist practices in healthcare delivery [48], particularly within oral health services, to build trust and ensure equitable access for Indigenous Communities. The inclusion of IHW in the oral health system is an opportunity to connect mainstream dental services and their Communities [7, 10, 11, 12, 13, 14]. However, several barriers hinder full participation of IHW in oral health promotion, including inadequate access to training opportunities and the persistent biases that continue to undermine their expertise. Addressing these challenges is imperative to empower IHW and enhance the effectiveness of oral health initiatives within Indigenous Communities [7]. Training IHW is more than skill‐building; it is an act of health sovereignty that restores power to Communities and addresses barriers created by colonial ideologies in mainstream systems.

The success of oral health training and programming hinges on active Community involvement, highlighting the critical importance of Community‐driven approaches, local leadership, and governance. Across the reviewed programs, Community involvement varied from consultation [35, 36, 37, 38, 42, 44] to local leadership [40, 41], governance [20, 39], and staffing [43]. The Ka'nisténhsera Teiakotihsnie breastfeeding program in Kanesatake, Quebec highlights how Community ownership and trusted local leadership drive health outcomes. By hiring a respected Elder, known as ‘Auntie’, to support new mothers, the program created a trusted, culturally resonant role that mothers preferred over traditional healthcare advice. This approach led to a significant rise in breastfeeding rates—from 19% to 75%—demonstrating the powerful impact of culturally grounded, Community‐driven support [49]. Similarly, a Community‐led oral health program for Aboriginal children in rural New South Wales, Australia included daily toothbrushing, water access initiatives, fluoride applications, and dental health education. The program achieved significant reductions in tooth decay, plaque levels, and gum inflammation, while increasing positive oral hygiene behaviours [50]. These examples consistently demonstrate that when Communities take the lead, outcomes improve significantly.

While knowledge is an essential foundation for oral health self‐determination, it alone does not change behaviours [51, 52]. We must go beyond mere training and actively implement strategies that redistribute power from colonial health systems to Communities so that sustainable oral health improvements can be achieved. Examples from Indigenous Communities, such as the First Nations Health Authority in British Columbia and the Kōkua Kalihi Valley in Hawai‘i, demonstrate this leadership. These health services reflect Community priorities, using holistic approaches that integrate physical, mental, emotional, and spiritual wellness. By embedding Community values and knowledge, these models create environments where health services resonate deeply with Community identity and support self‐determined health goals [53].

For sustainability and longevity among Indigenous Peoples globally, redistribution of power to self‐determined health services is essential. The examples above illustrate effective models, yet not all currently integrate comprehensive oral health. For example, in Australia, only a limited number of ACCHS are funded to provide dental services, and even those face challenges in attracting and retaining dental professionals [31]. Integrating a mandatory oral health component into the training of Aboriginal Health Workers and Practitioners (AHW/Ps) and Community Health Representatives (CHRs) could be a vital step toward embedding oral health more comprehensively within self‐determined health services. This approach would not only enhance the capacity of IHW to provide culturally appropriate oral health care, but also foster greater Community control over oral health outcomes. To support this shift, it is essential that funding is allocated for the development of specialised training and the delivery of this content by qualified trainers, ensuring that oral health is effectively incorporated into these programmes.

Indigenous health services disrupt the colonial limitations of mainstream health systems, providing trusted, culturally grounded care that respects communities identities and needs [54, 55]. These services offer more than just culturally safe care; they are clinically effective, as highlighted in the *Australian Health Review* (March 2017). ACCHS surpass other providers in improving Indigenous health outcomes [55]. They deliver comprehensive primary care tailored to clients' needs, including medical, allied health, public health, transport, child care and income support services [56]. To ensure these vital services can continue to deliver compassionate and equitable healthcare, they must receive robust funding and targeted oral health training. Only by channelling resources into these self‐determined health services can we dismantle colonial limitations and fully support the community‐centred approach to health that Indigenous peoples have already established and continue to lead.

This scoping review, to the best of our knowledge, is the first to focus on oral health promotion training specifically designed for IHW globally. A key strength of this review is its comprehensive approach, which includes grey literature and does not limit searches by language or region, thus aiming to minimise publication bias. However, limitations exist. Despite our global scope, only 11 programs from four countries were identified, highlighting the limited availability of published oral health training resources worldwide. Furthermore, this review does not capture knowledge conveyed in Indigenous languages or through oral traditions, which are often outside the scope of systematic searches. For this reason, traditional knowledge and narratives that are integral to Indigenous health contexts may not be fully represented.

## 5 Conclusion

Research consistently demonstrates that Indigenous Peoples experience significantly poorer oral health outcomes compared to the global population. Rather than perpetuating this cycle of highlighting disparities, we must shift our focus toward empowering Communities in exercising self‐determination over their oral (and overall) health. This transformation begins with the necessary training in oral health promotion and the allocation of sufficient resources and funding for Indigenous health services. By championing Indigenous leadership in health initiatives, we ensure that programs are deeply rooted in Community knowledge, values and priorities, laying the groundwork for sustainable improvements in health outcomes. Prioritising Community‐led initiatives not only restores ownership and cultural relevance to healthcare practices but also empowers Communities to reclaim decision‐making authority, fundamentally reshaping the healthcare landscape in a way that honours their sovereignty. Central to this shift is strengthening the IHW workforce through targeted training, which is crucial in enabling these workers to play an active role in oral health promotion and self‐determined health practices. This commitment to self‐determination is essential for dismantling the colonial frameworks that have historically marginalised Indigenous voices, paving the way for a future where Indigenous Peoples determine their health outcomes on their own terms.

## Conflicts of Interest

The authors declare no conflicts of interest.

## Supporting information


**Data S1:** cdoe70009‐sup‐0001‐supinfo.zip.

## Data Availability

The data that supports the findings of this study are available in the Supporting Information of this article.
