# Supplementary material for: Self‐Determination in Action: A Scoping Review on Oral Health Training for Indigenous Health Workers Globally
Source: Community Dent Oral Epidemiol. 2025 Aug 10;53(6):633–43. doi: 10.1111/cdoe.70009 (PMC12627271; doi:10.1111/cdoe.70009)
Supplement: Supplementary file 1 — Data S1: cdoe70009‐sup‐0001‐supinfo.zip. [file CDOE-53-633-s001.zip › Indigenous health workers oral health training-SystRev-LogicGrid.docx]

**Indigenous Health Workers & Oral Health: Scoping Review**

**PubMed**

Search Date: 4/06/2024

| Indigenous | Health Worker | Oral Health Promotion | Training |
| --- | --- | --- | --- |
| "first nation*"[tw] OR "pacific islander*"[tw] OR "torres strait islander*"[tw] OR aborigin*[tw] OR alaska*[tw] OR aleut*[tw] OR amerind*[tw] OR arctic[tw] OR aymara[tw] OR bushmen[tw] OR chukchi[tw] OR chukotka*[tw] OR circumpolar[tw] OR eskimo*[tw] OR greenland*[tw] OR indian*[tw] OR indigen*[tw] OR inuit*[tw] OR inupiaq[tw] OR inupiat[tw] OR khanty[tw] OR maori*[tw] OR mapuche[tw] OR metis[tw] OR native*[tw] OR navaho*[tw] OR navajo*[tw] OR nenets[tw] OR quechua[tw] OR sami[tw] OR tribal[tw] OR tribe*[tw] OR xingu*[tw] OR yup’ik[tw] OR yupik[tw] OR zuni[tw] OR "Oceanic ancestry group"[mh] OR "arctic regions"[mh] | "Community health work*"[tw] OR "Health work*"[tw] OR "Health aide*"[tw] OR "Health profession*"[tw] OR "family health*"[tw] OR "health service*"[tw] OR "Indigenous health work*"[tw] OR "Torres Strait Island* health work*"[tw] OR "Aboriginal health work*"[tw] OR "Aboriginal community controlled health se*"[tw] OR "Aboriginal medical service*"[tw] OR "public health*"[tw] OR "health care delivery*"[tw] OR "urban health service*"[tw] OR "Primary health care*"[tw] OR "Rural health service*"[tw] OR "Health care delivery*" [tw] OR "Aboriginal health service*"[tw] OR "First nation* health*"[tw] OR "Indigenous health service*"[tw] OR "Aboriginal community work*"[tw] OR "Indigenous community work*"[tw] OR "First nation* community work*"[tw] OR “workforce*”[tw] OR "community health service*"[tw] OR "traditional healer*"[tw] OR "health personnel*"[tw] OR "health* aide*"[tw] | “Dental health promotion” [tw] OR “Oral hygiene promotion” [tw] OR “Dental hygiene promotion” [tw] OR “Oral care promotion” [tw] OR “Dental care promotion” [tw] OR “Oral health education” [tw] OR “Dental health education” [tw] OR “Oral hygiene education” [tw] OR “Dental hygiene education” [tw] OR “Oral care education” [tw] OR “Oral health improvement” [tw] OR “Oral health campaigns” [tw] OR “Oral health interventions” [tw] OR “Oral health programs” [tw] OR “oral health promotion”[tw] OR “oral health”[tw] | “module”[tw] OR “training”[tw] OR “course*”[tw] OR “content”[tw] OR “seminar*”[tw] OR “lecture*”[tw] OR “tutorial”[tw] |

**Scopus**

Search Date: 4/06/2024

| Indigenous | Health Worker | Oral Health Promotion | Training |
| --- | --- | --- | --- |
| TITL-ABS-KEY "first nation" OR "first nations" OR "pacific islander*" OR "torres strait islander*" OR aborigin* OR alaska* OR aleut* OR amerind* OR arctic OR aymara OR bushmen OR chukchi OR chukotka* OR circumpolar OR eskimo* OR greenland* OR indian* OR indigen* OR inuit* OR inupiaq OR inupiat OR khanty OR maori* OR mapuche OR metis OR native* OR navaho* OR navajo* OR nenets OR Quechua OR sami OR tribal OR tribe* OR xingu* OR yup’ik OR yupik OR zuni OR "Oceanic ancestry group" OR "arctic regions"OR "Oceanic ancestry group" OR "arctic region" | TITL-ABS-KEY  "Community health work*" OR "Health work*" OR "Health aide*" OR "Health profession*" OR "family health*" OR "village health doctor*" OR "family planning personnel*" OR "village health work*" OR "health service*" OR "Indigenous health work*" OR "Torres Strait Island* health work*" OR "Aboriginal health work*" OR "Aboriginal community controlled health se*" OR "Aboriginal medical service*" OR "public health*" OR "health care delivery*" OR "urban health service*" OR "Primary health care*" OR "Rural health service*" OR "Health care delivery*" OR "Aboriginal health service*" OR "First nation* health*" OR "Indigenous health service*" OR "Aboriginal community work*" OR "Indigenous community work*" OR "First nation* community work*" OR workforce* OR "community health service*" OR "traditional healer*" OR "health personnel*" OR "medical provider*" OR "health* aide*" | TITL-ABS-KEY  “Dental health promotion” OR “Oral hygiene promotion” OR “Dental hygiene promotion” OR “Oral care promotion” OR “Dental care promotion” OR “Oral health education” OR “Dental health education” OR “Oral hygiene education” OR “Dental hygiene education” OR “Oral care education” OR “Oral health improvement” OR “Oral health campaigns” OR “Oral health interventions” OR “Oral health programs” OR “oral health promotion” OR “oral health” | TITL-ABS-KEY  “module” OR “training” OR “course*” OR “content” OR “seminar*” OR “lecture*” OR “tutorial” |

**Web of Science**

Search Date: 4/06/2024

| Indigenous | Health Worker | Oral Health Promotion | Training |
| --- | --- | --- | --- |
| "first nation*" OR "pacific islander*" OR "torres strait islander*" OR aborigin* OR alaska* OR aleut* OR amerind* OR aymara OR chukchi OR chukotka* OR eskimo* OR greenland* OR indian* OR indigen* OR inuit* OR khanty OR maori* OR mapuche OR metis OR native* OR navaho* OR navajo* OR nenets OR Quechua OR sami OR xingu* OR yup’ik OR yupik OR zuni OR "Oceanic ancestry group" OR "arctic regions"OR "Oceanic ancestry group" OR "arctic region" | "Community health work*" OR "Health work*" OR "Health aide*" OR "Health profession*" OR "family health*" OR "health service*" OR "Indigenous health work*" OR "Torres Strait Island* health work*" OR "Aboriginal health work*" OR "Aboriginal community controlled health se*" OR "Aboriginal medical service*" OR "public health*" OR "health care delivery*" OR "urban health service*" OR "Primary health care*" OR "Rural health service*" OR "Health care delivery*" OR "Aboriginal health service*" OR "First nation* health*" OR "Indigenous health service*" OR "Aboriginal community work*" OR "Indigenous community work*" OR "First nation* community work*" OR workforce* OR "community health service*" OR "traditional healer*" OR "health personnel*" OR "health* aide*" | “Dental health promotion” OR “Oral hygiene promotion” OR “Dental hygiene promotion” OR “Oral care promotion” OR “Dental care promotion” OR “Oral health education” OR “Dental health education” OR “Oral hygiene education” OR “Dental hygiene education” OR “Oral care education” OR “Oral health improvement” OR “Oral health campaigns” OR “Oral health interventions” OR “Oral health programs” OR “oral health promotion” OR “oral health” | “module” OR “training” OR “course*” OR “content” OR “seminar*” OR “lecture*” OR “tutorial” |

**EMBASE**

Search Date: 4/06/2024

| Indigenous | Health Worker | Oral Health Promotion | Training |
| --- | --- | --- | --- |
| ‘First nation*’ OR ‘pacific islander*’ OR ‘torres strait islander*’ OR ‘aborigin*’ OR ‘alaska*’ OR ‘aleut*’ OR ‘amerind*’ OR ‘arctic*’ OR ‘Aymara*’ OR ‘bushmen*’ OR ‘Chukchi*’ OR ‘chukotka*’ OR ‘circumpolar*’ OR ‘eskimo*’ OR ‘greenland*’ OR ‘indian*’ OR ‘indigen*’ OR ‘inuit*’ OR ‘inupiaq*’ OR ‘Inupiat*’ OR ‘Khanty*’ OR ‘maori’ OR ‘Mapuche*’ OR ‘metis’ OR ‘native*’ OR ‘navaho*’ OR ‘navajo*’ OR ‘nenets*’ OR ‘Quechua*’ OR ‘sami*’ OR ‘tribal*’ OR ‘tribe*’ OR ‘xingu*’ OR ‘yup’ik*’ OR ‘yupik*’ OR ‘zuni*’ OR ‘Oceanic ancestry group*’ OR ‘arctic regions*’ OR ‘Oceanic ancestry group*’ OR ‘arctic region*’ | 'rural health care’ OR 'rural health services' OR 'health personnel attitude' OR 'health care personnel' OR 'rural hospital' OR 'multidisciplinary care' OR 'health services' OR 'Aboriginal health worker*' OR 'Indigenous health worker*' OR 'community health worker*' OR 'first nation* health worker*' | ‘Dental health promotion’ OR ‘Oral hygiene promotion’ OR ‘Dental hygiene promotion’ OR ‘Oral care promotion’ OR ‘Dental care promotion’ OR ‘Oral health education’ OR ‘Dental health education’ OR ‘Oral hygiene education’ OR ‘Dental hygiene education’ OR ‘Oral care education’ OR ‘Oral health improvement’ OR ‘Oral health campaigns’ OR ‘Oral health interventions’ OR ‘Oral health programs’ OR ‘oral health promotion’ OR ‘oral health’ | ‘module’ OR ‘training’ OR ‘course*’ OR ‘content’ OR ‘seminar*’ OR ‘lecture*’ OR ‘tutorial’ |

**ProQuest Central**

Search Date: 4/06/2024

| Indigenous | Health Worker | Oral Health Promotion | Training |
| --- | --- | --- | --- |
| “First nation*” OR “pacific islander*” OR “torres strait islander*” OR “aborigin*” OR “indigen*” OR “maori” | "Community health work*" OR "Indigenous health work*" OR "Torres Strait Island* health work*" OR "Aboriginal health work*" OR "Aboriginal community controlled health se*" OR "Aboriginal medical service*" OR "Aboriginal health service*" OR "Indigenous health service*" OR "Aboriginal community work*" OR "Indigenous community work*" OR "First nation* community work*" OR "community health service*" OR "traditional healer*" | “Dental health promotion” OR “Oral hygiene promotion” OR “Dental hygiene promotion” OR “Oral care promotion” OR “Dental care promotion” OR “Oral health education” OR “Dental health education” OR “Oral hygiene education” OR “Dental hygiene education” OR “Oral care education” OR “Oral health improvement” OR “Oral health campaigns” OR “Oral health interventions” OR “Oral health programs” OR “oral health promotion” | “training” |
